# The Swedish Version of the eHealth Literacy Questionnaire: Translation, Cultural Adaptation, and Validation Study

**DOI:** 10.2196/43267

**Published:** 2023-04-12

**Authors:** Anna E Sjöström, Senada Hajdarevic, Åsa Hörnsten, Ólöf Kristjánsdóttir, Charlotte Castor, Ulf Isaksson

**Affiliations:** 1 Department of Nursing Umeå University Umeå Sweden; 2 Department of Public Health and Clinical Medicine, Family Medicine Umeå University Umeå Sweden; 3 Faculty of Nursing University of Iceland Reykjavik Iceland; 4 Department of Health Sciences Lund University Lund Sweden

**Keywords:** cultural adaptation, digital health, eHealth, eHLQ, eHealth literacy, health literacy, validation

## Abstract

**Background:**

With the increasing digitalization in health care, an effective instrument is necessary to assess health care consumers’ digital competencies—their “eHealth literacy.” The 7-scale eHealth Literacy Questionnaire (eHLQ), based on the theoretically robust eHealth Literacy Framework, has shown strong psychometric properties in Denmark and Australia.

**Objective:**

The aim of this study was to translate, culturally adapt, and evaluate the psychometric properties of the Swedish version of the eHLQ.

**Methods:**

We followed the Translation Integrity Procedure guidelines to translate and culturally adapt the questionnaire to Swedish using forward and backward translations, review by an expert panel, and cognitive interviewing. The psychometric properties of the Swedish eHLQ were investigated by evaluating its internal consistency (Cronbach α) and a priori–defined factor structure (confirmatory factor analysis).

**Results:**

A total of 236 primary health care patients and parents of hospitalized children were included in the validation analysis. The mean age was 48.5 years, and 129 (55%) were women. All 7 eHLQ scales showed good internal consistency, with the Cronbach α ranging from .82 to .92. Single-factor and 7-factor confirmatory factor analysis showed satisfactory model-fit values. With one exception, all items demonstrated satisfactory loadings on their respective factors.

**Conclusions:**

The Swedish eHLQ demonstrated strong psychometric properties. It has the potential as a useful tool for a variety of purposes, including population surveys, intervention evaluations, and eHealth service implementations.

## Introduction

Over the past decades, health care has undergone a rapid digital transformation. As health care services are increasingly delivered digitally, the internet has become a primary source of health information to people [1,2]. Sweden is considered a highly digitalized country, with 94% of all adult citizens reporting daily internet use and 80% reporting eHealth service use, including web-based health information acquisition, digital health care visits, and health application use [1]. The Swedish government has a vision that by 2025, Sweden will be the best country in the world at using eHealth services to make it easier for people to achieve good and equal health and to increase patients’ participation [3]. Digitalization benefits certain groups, but it might exclude others from using health care services and thereby increase health inequities [3]. The term “digital divide” was initially used in health care context to describe people’s unequal access to the internet and eHealth services, now usually refers to their unequal competencies to use such services: their eHealth literacy (eHL; also called electronic health literacy or digital literacy) [4].

Adequate eHL allows people to increase the availability of health information, which improves their health knowledge and leads to positive outcomes, including a better understanding of the medical condition, increased empowerment, more effective self-management, and better communication with health care professionals [5-7]. Furthermore, it has been shown to improve health-promoting behaviors, such as exercise and balanced nutrition, in certain patient groups [8,9]. During the COVID-19 pandemic, the importance of people’s eHL became evident as they needed to keep up to date with reliable web-based health information, be able to critically evaluate the veracity of web-based health information, and take part in eHealth services during the times of social isolation [10].

In 2006, Norman and Skinner [11] described eHL as “the ability to seek, find, understand, and appraise health information from electronic sources and apply the knowledge gained to addressing or solving a health problem.” However, the internet and eHealth systems have since undergone massive development, raising critiques of that definition for, among other things, disregarding interactive contexts (eg, social media) and situational, cultural, or social contexts [12,13]. Therefore, in 2015, Norgaard et al [14] used a validity-driven approach to capture the full range of elements relevant to the interaction between the individual and eHealth systems and developed the eHealth Literacy Framework (eHLF). By integrating the perspectives of a wide range of stakeholders, including patients, medical professionals, informatics professionals, and researchers, Norgaard et al defined 7 eHL domains, which are as follows: (1) ability to process health information, (2) engagement in own health, (3) ability to actively engage with digital services, (4) feel safe and in control, (5) motivated to engage with digital services, (6) access to digital services that work, and (7) digital services that suit the individual’s needs. The eHLF domains include necessary individual factors to use eHealth systems, eHealth system–relevant factors, and the interactional factors between the individual and the eHealth system [14].

The increasing use of eHealth services necessitates an effective instrument to assess health care consumers' eHL in areas of application, including population surveys, intervention evaluations, and eHealth service implementations [15]. Insight into people's eHL abilities is essential to properly deploy eHealth-related guidelines, strategies, and interventions [16]. Furthermore, this insight is crucial to, in accordance with the vision of the Swedish government, making eHealth services available and understandable to everyone who needs them [3,16]. Recent review articles have identified 8 available eHL instruments, with the eHealth Literacy Scale (eHEALS) the most frequently used by far, also in Sweden [17]. The eHEALS was developed using the original definition of eHL and simple health information technology, which do not correspond with current eHealth services [15,18,19]. As eHealth services have developed and become increasingly interactive over the last few decades, a more up-to-date eHL instrument is required in Sweden and elsewhere. The eHealth Literacy Questionnaire (eHLQ) is based on the 7 domains of the eHLF and was developed simultaneously in Danish (Denmark) and English (Australia). When tested in Denmark and Australia in large samples of the general population and people with chronic diseases, the eHLQ showed strong psychometric properties [20,21]. It is currently licensed for use in more than 12 countries, and its ongoing translations and cultural adaptations indicate that the instrument is robust across various contexts [20]. However, until now the instrument has not been translated into Swedish. The aim of this study was therefore to translate, culturally adapt, and evaluate the psychometric properties of a Swedish version of the eHLQ.

## Methods

### Study Design

This study used both qualitative and quantitative approaches. We first translated and culturally adapted the Swedish eHLQ and then psychometrically tested the adapted instrument.

### The eHealth Literacy Questionnaire

The eHLQ contains 35 items on 7 scales representing the eHLF domains: using technology to process health information, understanding of health concepts and language, ability to actively engage with digital services, feel safe and in control, motivated to engage with digital services, access to digital services that work, and digital services that suit individual needs. Each scale consists of 4 to 6 items on a 4-point Likert scale ranging from “strongly disagree” to “strongly agree.” Scale scores range from 1 to 4, calculated on an index by averaging item scores within each scale with equal weighting. Each scale is presented separately, and no overall eHLQ score is calculated. Higher scores indicate higher abilities [20].

### Translation, Cultural Adaptation, and Pretesting

License to translate the Danish version of the eHLQ to Swedish (02-2019) was obtained from Swinburne University, Australia. As required by the eHLQ developers, we used the translation integrity procedure (TIP) to maintain equivalence between the original (Danish) and translated (Swedish) versions of the instrument while ensuring the linguistic and cultural appropriateness of the Swedish version. The process was further facilitated by using clear “item intent” descriptions [22,23].

One of the eHLQ’s developers (Lars Kayser) chaired the TIP process. The translation and adaptation team included 2 native Swedish forward translators (ED, CC), 1 native Danish back translator (HG), a cognitive interviewer team (AS, UI), and 2 academic professionals (MP, OK), 1 fluent in both Swedish and Danish (MP) and both interested in local language and culture. All 3 translators had excellent Danish and Swedish language skills. The translation and cultural adaptation process involved three steps:

(1) The original Danish eHLQ questionnaire was translated independently into Swedish by the 2 forward translators. The translators then used the item-intent descriptions, which thoroughly explained the intent of each item and scale, as a guide when synthesizing their translations. During the following team discussion, the best statements for each item were chosen and combined to form the first version of the Swedish eHLQ.

(2) The first version of the Swedish eHLQ was back-translated by a native Danish-speaking translator, who had never seen the original version of the instrument. The Swedish-to-Danish back-translation was then compared with the Danish version of the eHLQ, and the items were discussed by the team to achieve consensus on the prefinal version of the Swedish eHLQ. (3) The prefinal Swedish eHLQ was then tested using cognitive interviews. Cognitive interviewing is valuable for ensuring accurate interpretations of items when translating and validating a questionnaire in another language and culture. This allows researchers to discover and correct items that are not interpreted as intended, thereby avoiding the future collection of inaccurate data. Cognitive interviewing does not require a large sample size, but the sample should represent a demographic variety [23,24].

Cognitive interviews were conducted with 9 adults (5 women) aged 18 to 80 (median 50) years, with varying educational backgrounds. The respondents were given a printed version of the questionnaire and were carefully observed while answering the items. The interviewer (AS) then went through each item with the individual respondents, focusing on items the respondent appeared to find difficult. The main questions were as follows: “What were you thinking about when you were answering that item?” and “Can you tell me why you selected that answer?” Participants were encouraged to elaborate on their interpretations of the items. A protocol was used for making notes during the interviews, which were also recorded, transcribed, and analyzed using a text summary [24].

Results from the cognitive interviews revealed that although most items were understood as intended, minor revisions were needed to clarify a few items and instructions. The following corrections were made throughout the Swedish eHLQ:

- The Swedish term digitala hälsosystem (digital health systems) was consistently replaced by digitala vårdtjänster (digital health care services) in items 9, 13, 16, and 28.
- The Swedish phrase Jag är säker på… (I am confident that…) was replaced by Jag känner mig trygg att… (I feel safe that…) in item 1.

When we reached an agreement on all formulations, the final version of the Swedish eHLQ was considered ready to be psychometrically tested.

### Data Collection

The data collection consisted of administering the Swedish eHLQ along with asking general questions about internet and eHealth service use. We also collected demographic data on participants’ age, sex, education, work situation, and health status. Because the topic of our study included digital use and literacy competencies, we chose to consistently administer paper-based questionnaires, although the eHLQ can also be administered on the web.

Data were collected from 2 rural and 2 urban primary health care centers (PHCs) in northern Sweden. Receptionists were asked to hand out questionnaires to all adults (≥18 years) Swedish-speaking patients visiting the PHC for 2 weeks in November 2020. Participants could fill out the questionnaire at the PHC or at home, returning it using an attached stamped and addressed envelope. A total of 178 questionnaires were collected from PHC patients.

An additional 64 questionnaires were filled out by parents of young children (<4 years) at the pediatric surgery or neonatal department of a hospital in southern Sweden. Nurses in the participating departments handed out questionnaires to Swedish-speaking parents of in-patient children in the spring of 2021. Otherwise, the procedure was identical to the PHC data collection.

### Statistical Analyses

Data analysis was performed using SPSS (version 25) and JAMOVI (version 2.2.3). Cases with ≥50% (n=18) missing values were excluded (n=2). Other missing values were replaced using the expectation––maximization algorithm imputation in SPSS. Demographic characteristics were reported by frequency and percentage (categoric variables) or mean and SD (continuous variables). Floor and ceiling effects were considered present if >15% (n=35) of participants reported the lowest or the highest response option for an item [25].

Because the aim of this study included the validation of a Swedish version of an instrument with scales that were defined a priori, our analyses were confirmatory. To evaluate internal consistency, Cronbach α was calculated separately for each scale. Internal consistency was considered good when Cronbach α was .70-.95 [25]. We also carried out confirmatory factor analysis (CFA). Initially, 7 single-factor models, one for each eHLQ scale, were fitted to the data to examine local independence by inspecting standardized factor loadings, modification indices, and standardized expected parameter change [26]. A strict 7-factor CFA was then performed, with no cross-loadings or correlated residuals. The diagonally weighted least squares (DWLS) estimator of the SEM (structural equation modeling) module in JAMOVI was used. The DWLS estimator is specifically designed for ordinal data such as Likert scales; it allows no distributional assumptions about the observed variables but assumes a normal latent distribution for each observed categorical variable [27]. Shi and Maydeu-Olivares [28] have suggested using standardized root mean residual (SRMR) for estimating model fit when using DWLS because other measures might be misleading. However, we also report the comparative fit index (CFI), Tucker-Lewis Index (TLI), root-mean-square of approximation (RMSEA), and chi-square (df) values. SRMR<0.09, CFI>0.95, TLI<0.95, RMSEA<0.05, and a chi-square/df (ie, chi-square value divided by the df) value of <3 indicated a close fit [29]. Factor loadings >0.40 were considered acceptable [30].

### Ethical Considerations

Ethical approval was granted by The Regional Ethical Review Board at Umeå University (no: 2014-179-31M) and The Swedish Ethical Review Authority (no. 2019-0341) and included a complementary application regarding expanded data collection. All steps were managed according to the General Data Protection Regulation and the ethical principles described in the Helsinki declaration [31,32].

## Results

### Demographic Characteristics of Participants

A total of 236 individuals completed the questionnaire, of whom 172 (73%) were PHC patients, and 64 (27%) were parents of hospitalized children. The sample included 129 (55%) women and 105 (45%) men aged 20-93 (median 48.5, mean 50.9) years; 130 (55%) worked, and 112 (48%) had a university education (see Table 1).

**Table 1:** Demographic characteristics (N=236).

			Total participants, n (%)		Patients (n=172), n (%)		Parents (n=64), n (%)
**Sex**							
	Male	105 (44.5)		77 (44.8)		28 (43.8)	
	Female	129 (54.7)		93 (54.1)		36 (56.3)	
	Prefer not to disclose	2 (0.8)		2 (1.2)		0 (0)	
**Age (year)**							
	≤35	78 (33.1)		34 (19.8)		44 (68.8)	
	36-55	53 (22.5)		33 (19.2)		20 (31.2)	
	56-74	61 (25.8)		61 (35.5)		0 (0)	
	≥75	42 (17.8)		42 (24.4)		0 (0)	
	Prefer not to disclose	2 (0.8)		2 (1.2)		0 (0)	
**Education**							
	Elementary school or less	28 (11.9)		26 (15.1)		2 (3.1)	
	Secondary school or vocational	96 (40.7)		75 (43.6)		21 (32.8)	
	University	112 (47.5)		71 (41.3)		41 (64.1)	
**Employment status**							
	Not working (unemployed or retired)	81 (34.3)		78 (45.4)		3 (4.7)	
	Working	130 (55.1)		70 (40.7)		60 (93.8)	
	Student	20 (8.5)		19 (11.0)		1 (1.6)	
	Other activity	5 (2.1)		5 (2.9)		0 (0)	

### Descriptive Statistics

The eHLQ mean scores ranged from 2.58 (SD 0.73) on scale 7 (digital services that suit individual needs) to 3.04 (SD 0.55) on scale 2 (understanding health concepts and language; see Table 2). The proportion of unanswered items ranged from 0.8% and 4.2%. The items with the largest proportion of missing values were items 26 (on scale 2) and 28 (on scale 7), both of which were left unanswered by 10 participants (4%; see Table 2). No ceiling or floor effects were detected at the scale level (not presented). Floor effects were detected in 3 items, whereas ceiling effects were present in most items. All items on scales 2, 3, and 4 demonstrated ceiling effects (see Table 2).

**Table 2:** Descriptive statistics for the Swedish eHLQ^a^ and analysis of floor and ceiling effects.

			Scale, mean (SD)		Item, median		Strongly disagree, n (%)		Disagree, n (%)		Agree, n (%)		Strongly agree, n (%)		Missing, n (%)
**Scale 1: using technology to process health information**			2.66 (0.73)												
	eHLQ7			3		20 (8.5)		33 (14.1)		89 (38.0)		92 (39.3)^b^		2 (0.8)	
	eHLQ11			3		25 (10.7)		54 (23.3)		94 (40.3)		60 (25.8)^b^		3 (1.3)	
	eHLQ13			3		26 (11.2)		67 (28.9)		108 (46.6)		31 (13.4)		4 (1.7)	
	eHLQ20			2		39 (17.0)^b^		90 (39.3)		84 (36.7)		16 (7.0)		7 (3.0)	
	eHLQ25			2		34 (14.8)		83 (36.2)		90 (39.3)		22 (9.6)		7 (3.0)	
**Scale 2: understanding of health concepts and language**			3.04 (0.55)												
	eHLQ5			3		10 (4.3)		28 (12.0)		140 (60.1)		55 (23.6)^b^		3 (1.3)	
	eHLQ12			3		9 (3.9)		29 (12.5)		113 (48.7)		81 (34.9 ^b^		4 (1.7)	
	eHLQ15			3		4 (1.7)		38 (16.5)		137 (59.3)		52 (22.5)^b^		5 (2.1)	
	eHLQ21			3		4 (1.7)		21 (9.2)		143 (62.4)		61 (26.6)^b^		7 (3.0)	
	eHLQ 26			3		15 (6.6)		48 (21.2)		119 (52.7)		44 (19.5)^b^		10 (4.2)	
**Scale 3: ability to actively engage with digital services**			2.90 (0.82)												
	eHLQ4			3		23 (9.8)		37 (15.8)		96 (41.0)		78 (33.3) ^b^		2 (0.8)	
	eHLQ6			3		17 (7.3)		37 (15.8)		102 (43.6)		78 (33.3)^b^		2 (0.8)	
	eHLQ8			3		32 (13.7)		48 (20.6)		91 (39.1)		62 (26.6)^b^		3 (1.3)	
	eHLQ17			3		22 (9.6)		50 (21.8)		94 (41.0)		63 (27.5)^b^		7 (3.0)	
	eHLQ32			3		23 (10.0)		58 (25.2)		97 (42.2)		52 (22.6)^b^		6 (2.5)	
**Scale 4** **: feel safe and in control**			3.03 (0.54)												
	eHLQ1			3		5 (2.1)		15 (6.4)		112 (48.1)		101 (43.3)^b^		3 (1.3)	
	eHLQ10			3		5 (2.2)		39 (16.9)		137 (59.3)		50 (21.6)^b^		5 (2.1)	
	eHLQ14			3		9 (3.9)		65 (27.9)		121 (51.9)		38 (16.3)^b^		3 (1.3)	
	eHLQ22			3		9 (3.9)		53 (22.9)		123 (53.2)		46 (19.9)^b^		5 (2.1)	
	eHLQ30			3		3 (1.3)		24 (10.5)		146 (64.0)		55 (24.1)^b^		8 (3.4)	
**Scale 5** **: motivated to engage with digital services**			2.64 (0.68)												
	eHLQ2			3		22 (9.4)		57 (24.5)		108 (46.4)		46 (19.7)^b^		3 (1.3)	
	eHLQ19			3		26 (11.3)		62 (27.0)		110 (47.8)		32 (13.9)		6 (2.5)	
	eHLQ24			2		37 (16.2)^b^		106 (46.5)		75 (32.9)		10 (4.4)		8 (3.4)	
	eHLQ27			3		26 (11.4)		69 (30.3)		98 (43.0)		35 (15.4)^b^		8 (3.4)	
	eHLQ35			3		18 (7.9)		51 (22.4)		105 (46.1)		54 (23.7)^b^		8 (3.4)	
**Scale 6** **: access to digital services that work**			2.70 (0.59)												
	eHLQ3			3		7 (3.0)		50 (21.7)		111 (48.3)		62 (27.0)^b^		6 (2.5)	
	eHLQ9			3		21 (9.1)		57 (24.6)		111 (47.8)		43 (18.5)^b^		4 (1.7)	
	eHLQ16			3		18 (7.9)		50 (22.0)		112 (49.3)		47 (20.7)^b^		9 (3.8)	
	eHLQ23			2		33 (14.5)		93 (41.0)		90 (39.6)		11 (4.8)		9 (3.8)	
	eHLQ29			3		35 (15.4)^b^		70 (30.8)		102 (44.9)		20 (8.8)		9 (3.8)	
	eHLQ34			3		21 (9.2)		55 (24.1)		124 (54.4)		28 (12.3)		8 (3.4)	
**Scale 7** **: digital services that suit individual needs**			2.58 (0.73)												
	eHLQ18			3		24 (10.6)		75 (33.0)		99 (43.6)		29 (12.8)		9 (3.8)	
	eHLQ28			2		32 (14.2)		86 (38.1)		94 (41.6)		14 (6.2)		10 (4.2)	
	eHLQ31			3		23 (10.0)		59 (25.8)		118 (51.5)		29 (12.7)		7 (3.0)	
	eHLQ33			3		27 (11.8)		64 (27.9)		102 (84.3)		36 (15.7)^b^		7 (3.0)	

^a^eHLQ: eHealth Literacy Questionnaire.

^b^>15% (n=35) of participants responded with the lowest or highest response option.

### Psychometric Properties

Cronbach α ranged from .82 to .92 (see Table 3). The single-factor CFA models generally showed satisfactory fit indices (see Table 3) and satisfactory to high factor loadings, ranging from 0.55 to 0.90, on 34 of 35 items. However, item 3 on scale 6, concerning whether information about the participant’s health is always available to those who need it, had a low factor loading (0.35). Nevertheless, all factor loadings were significant. The modification indices and standardized expected parameter change values (<0.2) revealed no correlated residuals, thereby supporting model fit.

A 7-factor model was then fitted to the 35 items. Considering the restricted model, with no cross-loadings or residual covariances allowed and a large number of items, the model fit was quite satisfactory (SRMR=0.06, CFI=1.00, TLI=1.00, RMSEA=0.00, *χ*^2^ divided by df=0.6). As with the single-factor CFA, all factor loadings were satisfactory to high except for item 3 on scale 6 (0.37; see Table 4).

Interfactor correlation coefficients in the 7-factor model ranged from 0.54 (scales 2 and 7) to 0.99 (scales 1 and 5). The second highest interfactor correlation coefficient, 0.97, was between subscales 6 and 7 (see Table 5).

**Table 3:** Cronbach α and model fit of the 7 single-factor models of the Swedish eHLQ^a^.

Scale	Cronbach α	SRMR^b^	CFI^c^	TLI^d^	RMSEA^e^	Chi-square divided by df (*df*)
1. Using technology to process health information	.88	0.04	0.99	0.98	0.04	1.3 (5)
2. Understanding of health concepts and language	.82	0.04	0.99	1.00	0.00	0.7 (5)
3. Ability to actively engage with digital services	.92	0.03	1.00	1.00	0.00	0.4 (5)
4. Feel safe and in control	.83	0.02	1.00	1.00	0.00	0.2 (5)
5. Motivated to engage with digital services	.87	0.04	1.00	1.00	0.00	0.8 (5)
6. Access to digital services that work	.82	0.04	1.00	1.00	0.00	0.9 (5)
7. Digital services that suit individual needs	.90	0.02	1.00	1.00	0.00	0.3 (5)

^a^eHLQ: eHealth Literacy Questionnaire.

^b^SRMR: standardized root mean residual.

^c^CFI: comparative fit index.

^d^TLI: Tucker-Lewis index.

^e^RMSEA: root mean square of approximation.

**Table 4:** Standardized factor loadings of the 7-factor model of the Swedish eHealth Literacy Questionnaire (eHLQ).

Scale and item		Factor loadings^a^ (95% CI)
**1. Using technology to process health information**		
	7	0.72 (0.67-0.77)
	11	0.78 (0.73-0.84)
	13	0.78 (0.73-0.84)
	20	0.69 (0.64-0.73)
	25	0.74 (0.69-0.79)
**2. Understanding of health concepts and language**		
	5	0.69 (0.62-0.75)
	12	0.76 (0.69-0.84)
	15	0.74 (0.68-0.81)
	21	0.56 (0.50-0.62)
	26	0.66 (0.59-0.72)
**3. Being able to actively engage with digital services**		
	4	0.87 (0.82-0.93)
	6	0.88 (0.83-0.94)
	8	0.71 (0.66-0.76)
	17	0.82 (0.77-0.87)
	32	0.87 (0.82-0.92)
**4. Feel safe and in control**		
	1	0.65 (0.59-0.71)
	10	0.74 (0.68-0.81)
	14	0.67 (0.61–0.73)
	22	0.70 (0.64-0.76)
	30	0.82 (0.75-0.88)
**5. Motivated to engage with digital services**		
	2	0.73 (0.68-0.78)
	19	0.82 (0.76-0.87)
	24	0.64 (0.59-0.69)
	27	0.74 (0.69-0.79)
	35	0.81 (0.76-0.86)
**6. Access to digital services that work**		
	3	0.37 (0.33-0.41)
	9	0.69 (0.64–0.74)
	16	0.72 (0.66-0.77)
	23	0.67 (0.62-0.72)
	29	0.66 (0.61-0.71)
	34	0.84 (0.78-0.89)
**7. Digital services that suit individual needs**		
	18	0.81 (0.75-0.86)
	28	0.76 (0.70-0.81)
	31	0.88 (0.82-0.94)
	33	0.86 (0.80-0.92)

^a^All loadings were significant at *P*<.01.

**Table 5:** Interfactor correlations coefficients among the 7 Swedish eHealth Literacy Questionnaire scales.

Scale	1	2	3	4	5	6	7
1. Using technology to process health information		0.73	0.88	0.60	0.99	0.74	0.77
2. Understanding health concepts and language			0.73	0.60	0.73	0.58	0.54
3. Being able to actively engage with digital services				0.58	0.87	0.75	0.73
4. Feel safe and in control					0.65	0.73	0.69
5. Motivated to engage with digital services						0.81	0.87
6. Access to digital services that work							0.97
7. Digital services that suit individual needs							

## Discussion

### Principal Findings

This study translated, culturally adapted, and evaluated the psychometric properties of the Swedish eHLQ. We used a systematic and rigorous translation and cultural adaptation process to reproduce the original instrument’s concepts and meanings. Our data from a diverse validation sample demonstrated that the Swedish eHLQ has strong psychometric properties and is in line with the psychometric outcomes of the versions in Danish and other languages [20,21,33].

As recommended for examining the validity of a translated instrument, this study used qualitative and quantitative approaches. During the first phase, we adopted TIP, which includes a multistep translation and review process and detailed item intent descriptions [22,23]. Results of the cognitive interviews and several review board meetings resulted in our revising a few words and phrases that were considered problematic in a Swedish context; however, most items on the Swedish eHLQ were understood as intended, and its equivalence to the original and translated versions was maintained [23]. The ease of translation and cultural adaptation could be attributed to similarities between the Swedish and Danish languages and cultures.

The proportion of unanswered items varied from 0.8% to 4%, indicating an acceptable item-response rate and a good understanding of the items. Our data revealed a few items with floor effects, but most items showed ceiling effects, which might indicate that a large proportion of our sample had confidence and trust in their abilities and the eHealth system [21]. A problematic aspect of the ceiling effect is that it might also indicate a lack of response options at the upper end of the scale, which could cause problems in distinguishing degrees of high ability among participants [25]. However, as discrimination between the most eHealth literate individuals is rare if ever the target of eHealth literacy research or eHealth interventions and implementations, this is not particularly relevant.

All scales demonstrated good internal consistency, with a Cronbach α of >.80. Consistent with Danish and Australian eHLQ validation studies, scales 2 and 6 showed the lowest values [20,21]. Our confirmatory factor analyses demonstrated the Swedish eHLQ to have a good psychometric structure, with each single-factor model and the 7-factor model showing satisfactory model fit. Most factor loadings were good, but item 3 on scale 6 (Having access to digital services that work) had a factor loading <0.40, which could indicate that it did not describe the factor well [34]. A possible explanation might be that this item concerns the respondent’s perception of health care professionals access to their health data while the other items on the scale concern the respondent’s own access to eHealth services. Consistent with the original Danish validity testing, the highest interfactor correlation coefficients were seen between factors 1 and 5 (0.99) and factors 6 and 7 (0.97), suggesting a potential lack of discriminant validity. Kayser et al [20], however, suggested that these high correlations may result from scales that are on the same causal path but that measure different constructs. This is theoretically supported by the underpinning robust framework that demonstrates strong content differentiation among the 7 domains of eHL [14,20].

Given our results, the Swedish eHLQ can be considered a good replication of the original instrument. The multidimensionality of this eHL instrument adds to the health literacy and eHL instruments currently available in Sweden. Although previous instruments such as the eHEALS have focused on individuals’ competencies, the eHLQ has the added perspective of the interaction between the individual and the eHealth systems [18,20]. Therefore, the Swedish eHLQ has the potential to be a valuable tool for various future purposes, including population surveys, intervention evaluations, and eHealth service implementations. Understanding health care consumers’ eHealth competencies and experiences of interactions with the eHealth system should enable eHealth system developers and health care providers to meet the needs of health care consumers and improve health equity.

### Limitations

Data collection was conducted during the first year of the COVID-19 pandemic, which created a considerable workload burden and a need among PHC and hospital staff to prioritize their tasks. The reduced number of PHCs able to participate and the shortened time available for data collection also reduced the intended sample size. Nevertheless, we decided our sample of 236 participants was appropriate, especially since the original eHLQ is a thoroughly designed and well-researched questionnaire [35,36]. Using both web-based and paper-based questionnaires could have increased the sample size. However, because our topic is concerned with internet use and health literacy competencies, we decided to prioritize consistency and simplicity, and therefore only administered paper-based questionnaires. However, web-based versions of the eHLQ should be tested in the future.

Our psychometric testing sample, which included primary health care patients and parents of in-patient children, represented a diversity of educational levels, genders, ages, and work statuses. Therefore, although it might not represent the national average, it reflected a generally diverse sample.

Another limitation is that eHealth–literate individuals might be overrepresented in this study because they are more likely to participate than people who consider themselves to have poor eHealth skills or who have negative attitudes toward eHealth service usage. This phenomenon is, however, difficult to avoid.

### Conclusions

This study suggests that the Swedish eHLQ is a reliable instrument, with good linguistic equivalence to the original and robust psychometric properties. Our sample consisted of PHC patients and parents of hospitalized children, but the questionnaire should be further tested in different demographic and disease groups. We expect the Swedish eHLQ will be a valuable tool for assessing multidimensional perspectives of eHL when conducting population surveys, intervention evaluations, and eHealth service implementations.

## Abbreviations

CFA: confirmatory factor analysis
CFI: comparative fit index
DWLS: diagonally weighted least squares
eHEALS: eHealth Literacy Scale
eHL: eHealth Literacy
eHLF: eHealth Literacy Framework
eHLQ: eHealth Literacy Questionnaire
PHC: primary health care center
RMSEA: root-mean-square of approximation
SRMR: standardized root mean residual
TIP: translation integrity procedure
TLI: Tucker-Lewis Index
